# From the parents’ perspective: qualitative exploration of caregiving challenges and coping strategies of children with intellectual disability

**DOI:** 10.1080/20473869.2025.2537119

**Published:** 2025-08-04

**Authors:** Ping Lu, Aizan Sofia Amin, Nor Jana Saim

**Affiliations:** a Centre for Research in Psychology and Human Well-being, Faculty of Social Sciences and Humanities, Universiti Kebangsaan Malaysia, Selangor, Malaysia; b Social Work Programme, Faculty of Social Sciences and Humanities, Universiti Kebangsaan Malaysia, Selangor, Malaysia

**Keywords:** Intellectual disability, parents, caregiving challenges, coping strategies, qualitative research

## Abstract

The care of children with intellectual disabilities (ID) is a complex and challenging process that deeply affects the lives and psychological of their parents. This study guided is by the Family Quality of Life (FQoL) theory and aims to understand the challenges faced by parents in caregiving and their coping strategies. This study employed a qualitative approach and conducted semi-structured interviews with 16 parents of children with ID. The interviews were recorded, transcribed, and translated into English. The data were analysed by thematic analysis. Parents reported the multidimensional impact of raising children with ID on the individual, family, and social levels and shared their coping strategies. Five themes emerged including emotional and psychological challenges, economic challenges, social challenges, children’s core disorder challenges, and coping strategies. Regarding coping strategies, parents adopted various strategies to overcome challenges including self-regulation, learning and growth, outdoor activities therapy, and seeking support. This study further explores the multifaceted challenges encountered by parents in raising children with ID and the effective coping strategies they employed. Moreover, the study offers valuable insights for policymakers, social workers, and educational institutions to develop more effective support systems for these families.

## Introduction

The global prevalence of intellectual disabilities (ID) is about 1% to 3% (Moeschler et al. 2014), and its occurrence is more prevalent in low- and middle-income countries (World Health Organization 2011). In developing countries, about 10 to 15 children out of every 1,000 are diagnosed with ID (Lee, Cascella, and Marwaha 2019), and China is no exception. According to the 2006s National Sample Survey on Disability, an estimated 82.96 million individuals in China were identified as having disabilities, comprising 6.34% of the total population. Among them, approximately 5.54 million were reported to have ID, accounting for 6.68% of the total disabled population (State Council Information Office of China 2006). As of 2020, the number of people holding disability certificates nationwide is 10.777 million, of which 864,000 hold ID certificates (China Disabled Persons’ Federation 2021).

According to the World Health Organization Report 2011, intellectual disability refers to a state of stagnation or incomplete mental development, which means that individuals may have difficulty understanding, learning, and remembering new things and may also face challenges in applying existing knowledge to new situations (World Health Organization 2011). The American Association on Intellectual and Developmental Disabilities also points out that intellectual disability is not just a low IQ (usually below 70 points) but also includes limitations in the adaptive skills that individuals demonstrate in daily life, such as self-care, communication, and social interaction (American Association on Intellectual and Developmental Disabilities 2021).

Intellectual disability may present as an isolated condition or co-occur with congenital anomalies or neurological disorders, including epilepsy, sensory impairments, and autism spectrum disorder (ASD). Its severity is typically categorised into mild, moderate, severe, or profound levels (Vissers, Gilissen, and Veltman 2016). Intellectual disability typically involves significant limitations in social (i.e. interpersonal interaction, self-identity), conceptual (i.e. time, language, and financial understanding), and practical (i.e. daily living tasks) skills (Lee, Cascella, and Marwaha 2019). These behaviours become more complex with age.

Studies have shown that parents who care for children with ID show significant psychological health and emotional overwhelm, they are more likely to feel frustrated and trapped and are more likely to experience anxiety and depression than ordinary family caregivers (Gogoi, Kumar, and Deuri 2017; Sharma et al. 2021). Parents who care for children with ID often face various difficulties and problems, including long-term care pressure, emotional burden, economic difficulties, and pressure from social prejudice (Özsavran et al. 2025). Most children with ID cannot complete basic life skills independently in daily life, so they need their parents to assist them with essential life care such as going to the toilet, eating, bathing, and dressing (Oti-Boadi 2017; Raliphaswa, Maluleke, and Netshikweta 2022). Moreover, most parents are apprehensive about their children’s future, especially how their children will live and take care of themselves after they die (Fernández-Ávalos et al. 2021). In addition, social prejudice and discrimination against children with ID exacerbate parents’ emotional distress and psychological stress (Raliphaswa, Maluleke, and Netshikweta 2022).

Chinese traditionalism and cultural introversion values (such as cultural superiority and intolerance of differences) are negatively correlated with attitudes towards people with ID (Hampton and Xiao 2009). Rooted in collectivist principles, Chinese culture prioritises social conformity over individual differences, which may lead to the perception that individuals with disabilities do not align with societal expectations, resulting in their exclusion and stigmatisation (Qu 2024). Additionally, in Chinese culture, children with disabilities are often a family’s secret (Huang, Kellett, and St John 2010), because people attach great importance to ‘face’, and giving birth to a child with ID is usually regarded as ‘losing face’. If people know that there is a child with ID in a family, it is a shame for the family (Chiu et al. 2015; Yang 2015). As a result, those who highly value social reputation may feel increased shame and adjust their behaviour to conform to societal norms. This shame and avoidance can deepen when expectations are unmet, such as having a child with ID, which is often seen as harming family honour (Yang 2015).

Parents need coping strategies to deal with the challenges of raising a child with intellectual disability. Coping is defined as an individual’s continuous efforts in thought and behaviour to cope with external or internal demands that are challenging or overwhelming (Folkman and Lazarus 1985). Studies have shown that parents mostly use emotional coping methods to deal with anxiety and depression (Sheikh et al. 2018), while some parents use meaning-centred coping strategies (Beighton and Wills 2017). A study in Brazil found that religion is an important psychological adjustment strategy used by mothers caring for children with ID (Rodrigues et al. 2019). In addition, Özsavran et al. (2025) showed that support from family members, friends, and medical staff helped mothers of children with ID overcome difficulties in parenting (Özsavran et al. 2025).

Research has pointed out that according to the findings of studies on children with autism and other developmental disabilities in China, familiar coping strategies for parents to cope with stress include acceptance, active coping, active reinterpretation and growth, and suppression of competing activities and plans (Shepherd et al. 2018; Wang, Michaels, and Day 2011). Su, Khanlou, and Mustafa (2021) also pointed out that parents with autism adopt Western views of autism to reduce stigma, set boundaries with grandparents, address unbalanced career dynamics, adjust authoritarian parenting styles, and expand social networks to cope with stress (Su, Khanlou, and Mustafa 2021). Other studies have also found that Chinese parents of children with disabilities maintain a positive attitude through family belief systems (such as destiny and living in the present), adaptability and cohesion, and build effective communication models to cope with parenting challenges (Chen and Yu 2024; Ma et al. 2023). However, currently there is limited study targeting the challenges and coping strategies of parents with ID in mainland China.

To better understand the caregiving challenges and coping strategies faced by parents of children with ID, this study adopted the family quality of life theory (FQoL) as a theoretical guide. FQoL is defined as a dynamic sense of well-being that is subjectively felt and determined by family members, in which the needs of individuals and families interact, evolve, and develop with continuous feedback loops (Zuna et al. 2010). The FQoL theory contains five core dimensions—emotional well-being, family interaction, parenting/care, material/physical well-being, and disability-related support—which provide a systematic perspective for a comprehensive exploration of the caregiving experience (Summers et al. 2005). These dimensions help to identify and understand the challenges faced by parents of children with ID in terms of emotional stress, family relationships, parenting burden, and resource acquisition, and their impact on family quality of life.

For example, Jenaro et al. (2020) found that the two dimensions of emotional well-being and disability-related support had a significant impact on the perceived stress of parents of children with ID (Jenaro et al. 2020), reflecting the theoretical value of these dimensions in the care context. Dizdarevic et al. (2022) pointed out, based on the FQoL scale, that the depression, anxiety, and stress levels of parents of children with ID are closely related to the overall quality of family life (Dizdarevic et al. 2022). This further supports the applicability of the FQoL theory in understanding the care stress of parents of children with ID.

Therefore, to gain a deeper understanding of the challenges faced by parents of children with ID in the process of care and their coping strategies, this study draws on the five core dimensions of the FQoL theory as a theoretical guide for analysing parents’ care experience. This will help social workers and other relevant professional institutions provide more comprehensive and practical support and services for parents of children with ID and help improve parents’ sense of well-being and quality of life.

## Materials and methods

This study used qualitative research methods. Qualitative research is defined as a method of exploring and understanding the meaning that individuals or groups attribute to social or human issues. It emphasises collecting data in natural settings, using inductive analysis, and using interviews and observations as research tools to gain a deep understanding of the participants’ experiences (Creswell and Poth 2016). Other studies also believe that qualitative research emphasises understanding phenomena in specific situations and interpreting them based on the meaning people give them (Cypress 2015). Therefore, this study explored the experiences of parents in their actual living environment through qualitative research to gain a deeper understanding of the multiple challenges they face - psychological, economic, social, children’s care related to ID, and to understand their adopted coping strategies to cope with these challenges.

According to Bryman (2012), semi-structured interviews are suitable for studying complex social phenomena owing to their flexibility and depth. They provide a framework to guide the interview direction, allow the order and content of questions to be adjusted, and dig deep into participants’ subjective experiences and opinions (Bryman 2012). Therefore, this study opted for semi-structured interviews. The interview questions were designed to align with the research questions, which focus on caregiving challenges and coping strategies. Interviews were recorded to be kept as an audit trail. The examples of questions such as: What was your reaction when your child was diagnosed with ID? What challenges do you face when caring for children with ID, and how do you cope with them?

### Participants

This study’s participants were 16 parents of children with ID who were selected from one of Social Work service agencies in Zhengzhou, China. The agency has provided services to more than 2,000 families of children with ID in Zhengzhou, including psychological counselling, resource links, and public education. It aims to help families cope with the multiple challenges they encounter in caring for their children.

Purposive sampling was used to select participants in this study until data saturation was reached. Parents of children with ID had to meet the following inclusion criteria to participate in the study:

- Participants had to be parents of children with ID,
- Parents involved in the daily care of children with ID,
- Parents with 2 years or more of caregiving experience,
- Parents can express themselves and communicate fluently.
- Parents are willing to participate in the study and agree to audio recording.

The participants’ ages ranged between 30 and 54, with an average of 40.94 years. Children with ID’s ages ranged between 6 and 18, with an average age of 11 years. All participants were married. Nine were employed, and seven were unemployed. Detailed information is provided in Tables 1 and 2, where Table 1 summarises the descriptive statistics of the parents and their children, and Table 2 lists the characteristics information of each participant and their children.

**Table 1.** Demographic characteristics of parents and their children.

	Parental characteristics	Children’s characteristics
Mean age	40.94 years, SD = 6.77	11 years, SD = 4.60
Age range	30–54 years	6–18 years
Gender	15 male, 1 female	14 male, 2 female
Mean caregiving duration	8.125 years, SD = 4.7	
Caregiving duration range	3–15 years	
No. of stay-at-home parents	9	
No. of employed parents	7	
Education level distribution	3 junior secondary, 4 high school, 1 associate degree, 6 bachelor, 2 master	
Marital status	16 married	

**Table 2.** Demographic information of parent participants and their children.

Participants	Gender	Age	Education level	Employment status	Gender of child	Age of child	Caregiving duration after diagnosis (years)
P1	Female	52	High school	Stay-at-home mom	Male	18	15
P2	Female	38	Bachelor	Stay-at-home mom	Male	8	6
P3	Female	54	Junior Secondary	Cleaner	Male	18	15
P4	Female	36	Master	Teacher	Male	9	6
P5	Female	41	High school	Stay-at-home mom	Male	14	11
P6	Female	36	Master	Financial manager	Male	10	7
P7	Female	30	Bachelor	Civil servants	Male	6	3
P8	Female	50	Associate degree	Kindergarten founder	Male	17	15
P9	Female	36	Bachelor	Teacher	Male	7	3
P10	Female	42	Junior Secondary	Stay-at-home mom	Female	7	4
P11	Female	33	Bachelor	Stay-at-home mom	Male	8	6
P12	Male	45	Bachelor	Civil servants	Female	6	3
P13	Female	45	High school	Stay-at-home mom	Male	8	6
P14	Female	38	Bachelor	Stay-at-home mom	Male	8	4
P15	Female	39	High school	Self-employed	Male	15	12
P16	Female	40	Bachelor	Teacher	Male	17	14

### Procedure

This study received assistance from one of Social Work Agency in Zhengzhou, China to recruit the research participants. The staff from the agency identify potential participants and explained the study to them. After initial contact, participants voluntarily and formally agreed to participate in the study. With the help from the Social Work Agency, the main researcher communicated with each participant by telephone to arrange the time and location for the interview to ensure maximum convenience and comfort. Some participants chose to be interviewed at home, while others preferred to be interviewed at the interview room in the Social Work Agency.

During the interview, the main researcher asked the participants to describe their caregiving experiences focusing on their challenges and coping strategies in raising children with ID. Data collection lasted for 3 months, and all interviews were conducted in Mandarin; one of dialects in Chinese language. The interviews were fully recorded using the recording function of mobile phones, ranging from 30 min to 1 h and 40 min.

### Ethical consideration

This study was approved by the Ethics Committee of the National University of Malaysia (Approval No: JEP-2024-661). All participants were fully informed about the study’s purpose and procedures and provided written informed consent before the interviews. Participation was entirely voluntary, and participants were informed of their right to withdraw at any time or decline to answer any question without consequence. To ensure confidentiality, pseudonyms were assigned and used throughout the study ensure participants’ privacy and data security.

### Data analysis

The data were analysed by using NVivo (version 15) software. This study followed the six steps of thematic analysis (Braun and Clarke 2006): (a) Familiarising data, (b) Generating initial codes, (c) Searching for themes, (d) Reviewing themes, (e) Defining and naming themes, and (f) producing the report.

During the data familiarisation process, all interviews were transcribed and translated to English by the main researcher. The translated verbatim were then back translated to ensure accuracy and fidelity to the original meaning. Next, the researchers carefully reviewed the transcriptions to identify keywords or phrases related to the challenges faced by the interviewees and their coping strategies. Subsequently, the core themes were extracted by marking and classifying the relevant content. Finally, the core themes were clarified by grouping the relevant content, and further sub-themes were formed.

According to Yin (2009), credibility refers to the fact that the operational process of the research (such as data collection procedures) can be repeated and produce the same results (Yin 2009). Creswell and Miller (2000) emphasised the use of a variety of methods (such as triangulation, member checking, audit trails, etc.) to enhance the credibility of the research. Among them, peer debriefing is regarded as one of the effective means (Creswell and Miller 2000). Therefore, this study’s credibility was enhanced through peer debriefing and an audit trail, in which two peers with extensive qualitative analysis experience participated in the discussion of emerging themes. Complete audit records were kept, ensuring the transparency and verifiability of the data analysis process.

## Results

**Figure 1.**
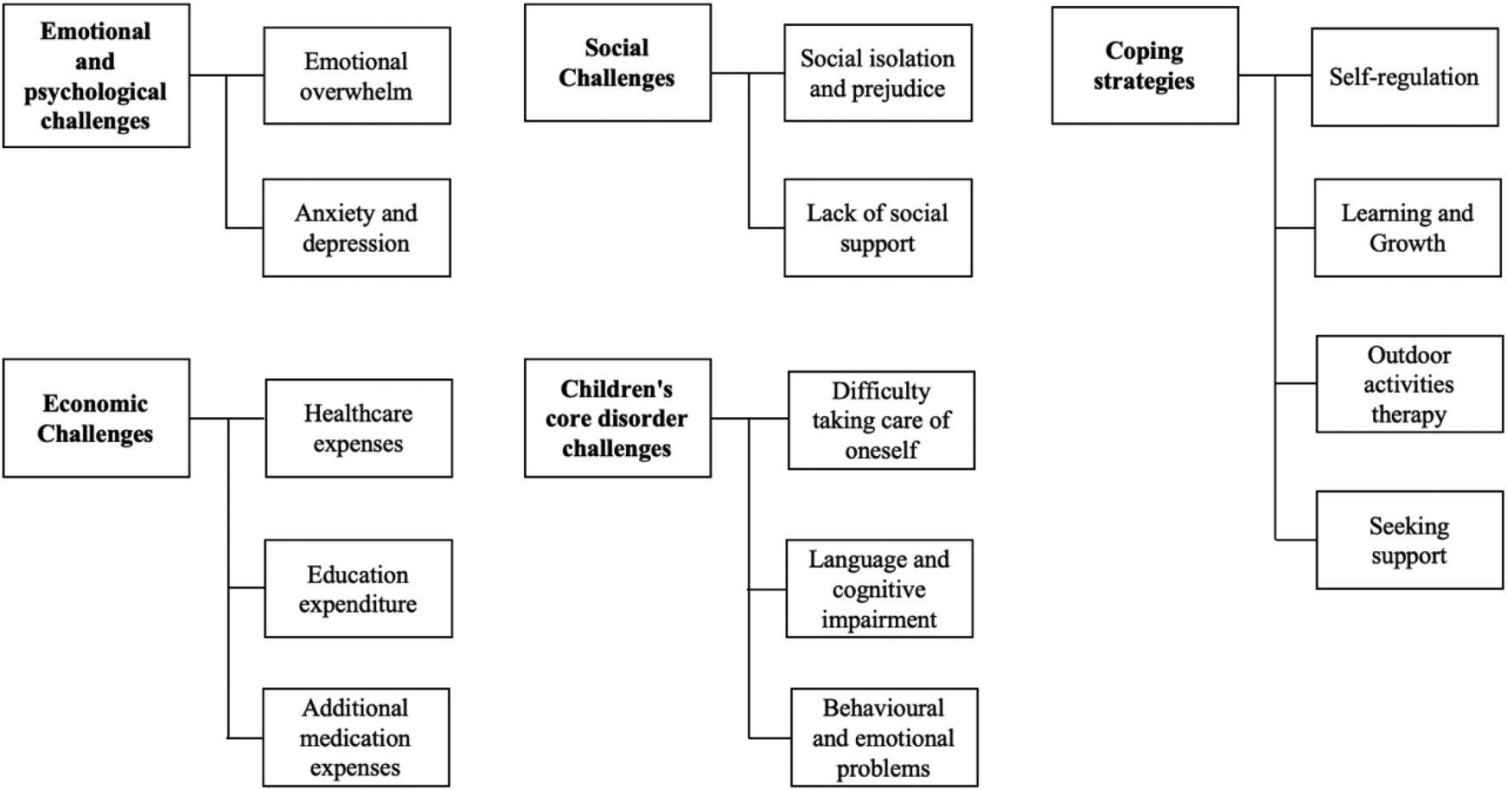
Main themes and sub-themes.

Through the analysis of interview transcripts, five main themes emerged: (a) emotional and psychological challenges, (b) economic challenges, (c) social challenges, (d) children’s care disorder challenges, and (e) coping strategies. These themes comprehensively reflect the challenges faced by parents of children with ID in the caregiving process and the strategies they employ to cope. Each main theme encompasses specific sub-themes, which are detailed in Figure 1.

### Theme 1: Emotional and psychological challenges

Parents of children with ID usually experience a strong psychological shock when they are diagnosed, including extreme sadness, inability to accept, shock, and confusion. In the long-term process of accompanying their children to grow up, parents’ emotional reactions are further complicated, and anxiety and depression are common psychological stress.

#### Emotional overwhelm

Parents reported experiencing a range of intense emotions while caring for children with ID, including sadness, denial, shock, distress, frustration, feelings of inferiority, avoidance, and concern. They experienced profound sadness upon first learning of their children’s intellectual disability diagnosis. Some parents even lost emotional control, succumbing to deep sorrow, breaking down in tears, and struggling to come to terms with this reality. A parent recounted this loss of emotional control:

When I learned that my child was diagnosed with intellectual disability, I felt utterly devastated and cried. Because I cried too much, my eyes got dry syndrome. I still feel sad and difficult to accept when I think about it now. (P4)

In addition to profound grief, parents also experienced shock and confusion—shock as an instinctive response to unexpected news, and confusion from not understanding the cause of their child’s diagnosis. For example, one mother stated, ‘When my child was first diagnosed, I was in shock and thought the hospital was wrong’. Another mother described a similar experience:

When my child was first diagnosed, I was extremely shocked and difficult to accept. Now, I still struggle to understand why this happened, as my family has had no history of such conditions for generations. This sense of confusion has continued to affect me. (P15)

Many parents also feel inferior and frustrated because of their children’s intellectual disability, especially those with higher social status or better economic conditions. For example, a mother was a financial manager in a company. She had long believed that she was in a relatively superior social position. However, her child’s low intelligence made her feel inferior, and she thought this situation made her inferior to others. Similarly, another parent stated:

As a leader in the unit, I always thought I had a certain status and influence in society. However, when my child was diagnosed with intellectual disability, I felt very inferior and felt that children with intellectual disabilities were failures, which made me feel deeply frustrated. (P12)

Furthermore, some parents avoid discussing their child’s intellectual disability due to fears of social discrimination, as they perceive it as a source of shame and believe it causes them to ‘lose face’ in society. For example, one mother shared:

I am terrified that others will know that my child has an intellectual disability. I will feel embarrassed and ‘lose face’. I am also worried that I will be discriminated against, so I will not take the initiative to talk to others about my child’s situation. (P14)

Many parents also reported concerns about their children’s future. They were particularly concerned about not being able to continue to care for their children as they aged, or their health changed. For example, one mother described:

I am worried that if I get sick or die one day, who will take care of my child? Moreover, as I get older, I find it more and more difficult to take care of my child. (P6)

Another mother shared how worried she was about her child’s future employment. She hopes her child can develop basic job skills, achieve a sense of self-worth through work, and reduce their dependence on the family. The mother emphasised:

I am deeply concerned about my child’s future employment. I hope he can find a stable, safe, and basic job in the future so that he can feel valuable and less dependent on the family. (P13)

#### Anxiety and depression

Conversations with parents revealed that anxiety and depression are common psychological states for many parents when dealing with children’s special needs for a long time. For example, one mother said that her child’s picky eating or refusal to eat made her feel anxious and depressed. She elucidated further on this topic:

My child’s eating problem is my biggest headache. He is either extremely selective or refuses to eat altogether. Each day, he only consumes a very limited variety of foods, leading to inadequate nutrition. This has caused me significant anxiety and even depression. (P4)

Furthermore, all parents said that they invested a lot of time and energy in the hope that their children would make significant progress in rehabilitation, but when the rehabilitation effect was not ideal, they felt anxious and depressed. For example, one mother described her emotions as follows:

Whenever I see other children in the same program making rapid progress while my child improves much more slowly, I feel extremely anxious and experience intense self-doubt, wondering if my supervision was insufficient and hindered my child’s learning progress. (P1)

### Theme 2: Economic challenges

Parents in this study reported that healthcare expenses, education costs, and reduced income have caused severe economic challenges for their families. Children’s rehabilitation, shadow teachers, additional courses, and private school tuition fees bring high expenses, and some families also need to buy long-term medicine for their children. Additionally, many parents have to resign from their jobs to care for their children, further exacerbating the economic challenges.

#### Healthcare expenses

Parents indicated that the high cost of rehabilitation for children with ID has led to severe economic challenges for their families. For example, a mother expressed that her child was treated in a private rehabilitation institution, which was expensive. Three to four months of rehabilitation cost 80,000 to 90,000 yuan (approximately USD 11,200 − 12,600), which caused her family to face economic challenges. Another mother expressed a similar experience:

My child has been receiving rehabilitation at a private institution, the monthly rehabilitation expenses amount to approximately 25,000 yuan (approximately USD 3,500). Over the past six years, the total cost of his rehabilitation has reached nearly 1.5 million yuan (approximately USD 210,000), leaving my family without any savings. (P11)

In addition to rehabilitation costs, some parents reported that their children’s emotional problems, psychological issues, and sleep disorders necessitate long-term medication use, contributing to economic challenges. For example, one mother stated:

My child has to take several medications every day to treat his emotional and sleep problems. These medications are very expensive. Almost all his father’s and my salaries are spent on them. (P3)

#### Education expenditure

To ensure their children can smoothly integrate into the school environment, some parents need to hire shadow teachers and enrol their children in additional courses to enhance their abilities. However, the high cost of these services poses significant economic challenges for families. For example, a mother described her experience:

To prevent my child from affecting others at school, I hired a shadow teacher with a monthly salary of 7,000 yuan (approximately USD 980). Additionally, my child has relatively poor social skills, so I enrolled him in extra social skills training classes, for 400 yuan per lesson (approximately USD 56). As a result, my family is heavily in debt. (P6)

In addition to paying for additional shadow teachers and courses, private school education expenses also cause parents economic difficulties. One mother explained that due to the severity of her child’s obstacle degree, the public inclusive primary school refused to accept her child. Her child could only attend a private primary school with an annual tuition of up to 30,000 yuan (approximately USD 4,200), which saddled her family with a heavy economic burden. A parent also shared the following experience:

My child is currently enrolled in an inclusive kindergarten, where the monthly tuition is 4,000 yuan (approximately USD 560), whereas the tuition for children in regular kindergartens is only 2,000 yuan (approximately USD 280). Her tuition is twice that of typically developing children. (P12)

#### Reduced income

Some parents in the study reported having to leave their jobs to care for their children with ID. Even for those who managed to continue working, their caregiving responsibilities often negatively impacted their professional performance. One mother, through tears, shared her experience:

Before my child was diagnosed, I had a well-paid job. However, after my child was diagnosed with intellectual disability, I had to quit my job and accompany him in his rehabilitation training, which directly led to a significant reduction in my family’s income. (P13)

One of the participants also talked about the negative impact of the birth of a child with ID on his job:

The birth of my child has had a great impact on my job. I may be promoted to a higher position if I hadn’t had this child. However, now that I have to take care of my child with intellectual disabilities, I don’t have as much time and energy to focus on my work, so I can only stop in my current position. (P12)

### Theme 3: Social challenges

Parents reported social isolation, prejudice, and insufficient support while caring for children with ID. Some parents lost social connections and faced strained family relationships. Limited government support in education and medical further intensified parent’s caregiving challenges.

#### Social isolation and prejudice

Some parents reported that their lives became entirely centred around their children, leading to feelings of social isolation and the gradual weakening of family bonds. One mother, overcome with emotion, tearfully recounted her experience:

To take care of my child, I almost had no time to interact with relatives and friends. I was busy with rehabilitation and tutoring my child every day, and my social circle was limited to the families of children with ID. I felt that I was eliminated by society. (P5)

Additionally, some parents reported experiencing social prejudice against both their children with ID and their families. This prejudice was evident in the misinterpretation and stigmatisation of their children’s behaviours, as well as the neglect and misunderstanding faced by their families. One mother shared her experience:

I remember one time when I took my child to a public place, he suddenly screamed. A passerby shouted at me angrily: ‘Is your child a fool? Can you let him stop screaming?’ At that moment, I felt very sad and deeply felt the misunderstanding from society. (P4)

#### Lack of social support

Some parents reported experiencing significant challenges in the daily care of their children with ID. These challenges were primarily attributed to insufficient informal support, particularly from their partners and other family members, contributing to increased tension within family relationships. One mother shared her perspective:

The child’s daily life and rehabilitation are completely taken care of by me alone. The child’s father, grandfather, and grandmother never help to take care of him. This also caused a strain on my relationship with my family. We quarrelled constantly. (P11)

Furthermore, some parents reported that the government’s inadequate educational and medical support increased their caregiving challenges. For instance, one mother (P10) explained that her child was unable to enrol in a public primary school due to household registration restrictions, exacerbating her caregiving difficulties. Another mother reflected on the government’s rehabilitation subsidies stated:

The rehabilitation training methods of the three government-subsidized hospitals are not suitable for my child. Consequently, I had to take my child to a private institution for rehabilitation treatment, which is highly expensive and places a significant burden on my family. (P7)

### Theme 4: Children’s core disorder challenges

Parents said that intellectually disabled children have difficulties in self-care, language cognition, and behavioural emotions, which poses significant challenges to parents.

#### Difficulty taking care of oneself

Parents reported that they must provide comprehensive assistance to their children in performing daily activities, including eating, brushing their teeth, bathing, dressing, and using the toilet. A parent expanded further on this issue:

I have to assist him in eating, bathing, going to the toilet, and dressing every day. For example, he often wears his clothes backwards, and I need to constantly teach him the correct way to dress over and over again. Repeating these trivial matters for a long time has worn out my patience. (P13)

#### Language and cognitive impairment

Some parents reported that their children’s delayed development in language and cognitive abilities necessitates continuous repetition and guidance, thereby increasing the challenges associated with caregiving. The following mother shared her experience:

My child is now eight years old, but his language ability is still at the level of a three-year-old. For example, when I was teaching him, ‘I want to go to the toilet’, I had to teach him each word separately and then slowly put it together into a sentence. It took him a long time to master it. (P2)

#### Behavioural and emotional problems

Some parents reported their children’s behavioural and emotional problems, such as crying, running around in public, or losing control of their emotions at home. These behavioural problems brought significant care challenges to parents, making them feel ashamed, embarrassed, and worried about affecting others. For example, one mother explained that his child often cried, screamed, and ran around uncontrollably in unfamiliar public places, which made her feel very ‘faceless’ and embarrassed. Another mother also described such feelings:

Sometimes, my child would have an emotional outburst at night and would not sleep. Although I tried my best to comfort him, he would still jump loudly and even stomp on the floor to vent. I would feel ashamed and worried that these bad behaviours of the child would affect the neighbours. (P7)

### Theme 5: Coping strategies

Parents reported employing various coping strategies to manage the challenges associated with caring for children with ID. These strategies included self-regulation, learning and growth, outdoor activities therapy, and seeking support.

#### Self-regulation

Some parents consider self-regulation to be the most effective strategy for managing stress and maintaining a positive mindset. They perceive external support as limited, emphasising the importance of internal adjustment. One mother explained that as a parent of a child with ID, the inability to self-regulate could lead to persistent mental and psychological challenges. Consequently, she regards internal self-regulation as the most effective coping strategy. Another mother also shared her approach to self-adjustment:

When I no longer compared him with normal children and did not regard his growth as a “battle,” I learned to accept his pace. After an internal adjustment, I felt less stressed. (P2)

#### Learning and growth

Some parents manage the challenges associated with caring for children with ID through continuous learning. Their learning encompasses various topics, including intervention strategies for children with ID, psychological stress management, and classical literature. The following statement from two mothers illustrate this perspective:

I like to read some chapters in the Analects and find my direction from the teachings of the ancients. Confucius said, ‘The wise are not confused, the benevolent are not worried, and the brave are not afraid’, which made me understand that I should learn to solve problems with wisdom and treat children with benevolence, which is my spiritual support on the road of parenting. (P15)

I enrolled in several paid online courses conducted by international mothers with experience in caring for children with intellectual disabilities. Every day after my child fell asleep, I would watch the material of the courses and learn relevant intervention knowledge. At the same time, I also bought lots of books on psychological decompression and learned ways to relieve stress. (P7)

#### Outdoor activities therapy

Some parents mentioned that they often use outdoor sports to solve challenges in the parenting process. For example, walking, climbing, and running allow parents to release the accumulated psychological stress, temporarily withdraw from busy care, and gain physical and mental relaxation. The following mother illustrates this perspective:

I enjoy taking my child mountain climbing or playing badminton in open spaces. Initially, my goal was to ensure that my child got enough exercise. However, I later realized that these activities also serve as a form of relaxation for me. (P6)

#### Seeking support

Some parents seek emotional support by engaging in activities organised by social work agencies or by communicating with family, friends, and colleagues. These activities provide temporary relief from the stress of caregiving and promote physical and mental relaxation. For example, one mother shared that participating in parent respite activities, such as pottery and embroidery, allowed her to escape anxiety momentarily, focus on creativity, and experience a sense of accomplishment. Another mother found comfort, warmth, and support through conversations with friends.

I often talk to a former colleague. Once, I could not help crying, she comforted me and said, ‘All difficulties will pass. If you need help, I will do my best to help you.’ At that moment, I felt hot. (P13)

In addition, some parents mentioned that although psychological counselling services require a certain amount of economic investment, they will still actively seek help from professional psychologists to obtain psychological relief and guidance on coping strategies. A mother shared her experience of receiving continuous psychological counselling:

My child has a severe intellectual disability, and he often has various behavioural problems. In addition, I have been taking care of him alone for many years, which is too tiring. I have a fixed psychologist. Whenever I feel depressed, I will consult him for consultation and treatment, but this requires payment. (P16)

## Discussion

This study explored the caregiving challenges experienced by Chinese parents of children with ID and their corresponding coping strategies. The findings demonstrate that these challenges do not exist in isolation but are interconnected across multiple core dimensions of FQoL theory. Specifically, challenges including emotional and psychological, economic, social, and children’s core disorder reveal the vulnerability of families in terms of emotional and material well-being, family interactions, disability-related support, and parenting function. At the same time, parents’ proactive efforts to address coping strategies, such as learning intervention knowledge, self-regulation, and seeking support, reflect their efforts to cope with multidimensional challenges, reduce stress, and improve family quality of life. These findings underscore the theoretical relevance and cross-cultural applicability of the FQoL theory in understanding the lived experiences of caregivers of children with ID in the Chinese context (Hu, Wang, and Fei 2012).

The emotional and psychological challenges of parents when their children are diagnosed with ID align closely with previous research findings, including emotional overwhelm during the initial diagnosis stage and ongoing psychological stress in long-term caregiving. Studies have shown that upon receiving a diagnosis of intellectual disability, parents often experience emotions such as grief, sadness, denial, shock, and anger (Fernández-Ávalos et al. 2021; Oti-Boadi 2017). These emotional reactions and psychological stress are closely related to the psychological/emotional well-being dimension in FQoL theory. Studies have shown that low emotions and psychological stress significantly impact the family quality of life of parents of children with ID (Cheng et al. 2025). Additionally, parents of children with ID are at a higher risk of developing anxiety, depression, or both throughout long-term caregiving (Sharma et al. 2021). Furthermore, some parents may experience feelings of inferiority and ‘lose face’ due to having a child with an intellectual disability, leading them to avoid discussing their child with others (Khalil Arjmandi, Kashani Vahid, and Vakili 2024; Yang 2015).

The parents in this study expressed concerns about who would care for their children with ID in the event of their illness or death. It is common for parents of children with ID to worry about the uncertainty of their children’s future, particularly regarding their survival and caregiving arrangements, if they become unable to provide care (Oti-Boadi 2017). Additionally, this study found that parents hope their children with ID will secure suitable employment as they grow older. Previous research has similarly indicated that parents of children with ID aspire for their children to achieve independence, sustain themselves financially, and obtain a job in the future (Niedbalski 2020).

Parents reported facing significant economic challenges while raising children with ID, particularly due to the costs associated with their children’s education, medical care, and rehabilitation. Additionally, their employment and income were negatively affected, leading to a reduction in overall family earnings. Existing research indicates that raising a child with ID imposes substantial economic burdens on families, governments, and society, including expenses related to medication, rehabilitation, education, and productivity losses (Shahat and Greco 2021). Furthermore, parents caring for individuals with ID often struggle to find employment despite most expressing a desire for paid work (Ejiri and Matsuzawa 2017). A study pointed out that the family’s income and economic status are important components affecting the family’s well-being and FQoL (Luitwieler et al. 2021). This shows that the family’s economic status is important to the parents’ economic well-being and overall quality of life.

In this study, parents reported experiencing social prejudice and exclusion. Previous research has similarly found that social exclusion, bullying, and stigmatisation are common experiences for children with ID and their families (Collings and Llewellyn 2012). Mothers who care for children with ID often face reduced social engagement due to the high intensity of caregiving responsibilities and emotional stress, leading to social isolation (Özsavran et al. 2025). Additionally, some parents highlighted issues related to social stereotypes, which primarily involve stigmatisation and negative perceptions of children with ID. These stereotypes including the belief that children with ID cannot communicate or understand, are ‘difficult’ patients, and that their parents are problematic or ‘unqualified’ (Aston, Breau, and MacLeod 2014). These stigmatised identities and perceived discrimination have significant negative impacts on parents’ well-being (Ye, Sin, and Gao 2021).

The lack of support from family members or service agencies exacerbates the caregiving challenges faced by parents. Many parents report feeling isolated and helpless (Chang and McConkey 2008). Consequently, most families of children with ID seek various forms of support, including informational, emotional, and financial assistance, as well as government policy adaptations and access to residential facilities (Lahaije et al. 2024). Parents’ desire for support systems reflects deficiencies in the disability-related support dimension. This dimension is an important predictor of FQoL, highlighting its key role in enhancing family well-being (Alnahdi 2024).

Parents in this study also discussed the daily care needs of children with ID, which significantly increases parental caregiving challenges. They must assist their children with all aspects of daily living, including eating, brushing teeth, bathing, dressing, and using the toilet (Oti-Boadi 2017; Raliphaswa, Maluleke, and Netshikweta 2022). Additionally, the study found that children’s behavioural problems are one of the sources of care challenges for parents. Common problems include violent destruction, inappropriate social interaction, self-harm, hyperactivity, temper tantrums, screaming, and getting lost, which have a profound impact on the family’s daily life (Chang and McConkey 2008). Furthermore, parental caregiving challenges are closely linked to the severity of their children’s cognitive and communication impairments (Jung et al. 2023). Those high care needs and challenging behaviours of children with ID significantly raise parental stress, and reducing family quality of life (Staunton, Kehoe, and Sharkey 2023).

When faced with the challenges of caring for children with ID, parents primarily cope through self-regulation, encouraging themselves to maintain an optimistic attitude. Heiman (2002) also pointed out that parents of children with ID emphasise the importance of having firm confidence in their children’s future, maintaining a positive outlook on life, and accepting the realities of disability (Heiman 2002). In addition, positive beliefs can bring higher social acceptance and social resources, and improving family happiness, welfare, and resilience (Gao, Lu, and Drani 2023). However, excessive positive beliefs may have a counterproductive. During the interview, one mother shared that her husband refused to accept the child’s intellectual disability and placed unrealistic expectations on the child, leading to stress, strained parent-child relations, and harm to family well-being.

Additionally, some parents seek to alleviate their emotional distress by improving their knowledge and skills through learning. This includes studying traditional culture and reading books on parenting and psychological stress management. Research has shown that traditional cultural philosophies, particularly Taoism and Confucianism, promote mental well-being by advocating the regulation or transformation of personal desires and emphasising harmony between individuals, nature, and society (Wang 2022). Furthermore, some parents acquire practical skills such as caregiving and psychotherapy by searching for resources online, reading books on parenting and stress management, advocating for their children, navigating support systems, and providing direct care (Matthews, Puplampu, and Gelech 2021). However, although self-education can help parents relieve stress and improve coping skills, they may still face obstacles, such as high treatment costs, childcare arrangement issues, and the availability of professional service providers (Osborn, Roberts, and Kneebone 2020).

In addition, some parents engage in outdoor activities as a means of relaxation. Sheikh et al. (2018) found that most parents cope with anxiety and depression through self-distraction, behavioural detachment, and emotional venting (Sheikh et al. 2018). Cardoso, Lumini, and Martins (2025) also noted that physical exercise interventions can reduce caregivers’ stress and improve parents’ overall well-being (Cardoso, Lumini, and Martins 2025). This study also found that parents actively seek various forms of support, including professional, peer, psychological, and financial assistance, to help them navigate caregiving challenges (Lahaije et al. 2024). Social support established through proactive intervention by parents of children with ID can improve parents’ well-being (Halstead et al. 2017). However, despite the positive effects of this strategy, it also comes with a particular cost burden. For example, a study found that parents caring for children with disabilities had significantly higher rates of mental health service use and related costs than ordinary families (Chen et al. 2023).

## Strengths and limitations

The strengths of this study are that it focuses on the caregiving experiences of parents of children with ID in China, filling the gap in previous studies that mainly focused on families of a wider group of children with disabilities or special needs. In addition, this study reveals the challenges parents face in the care process and explores the coping strategies they demonstrate, helping to break the stereotype that ‘caregivers are sufferers’. Moreover, this study offers a reference for future research, especially in Chinese society and culture, and provides valuable inspiration for improving the quality of family life of parents of children with ID.

However, this study has certain limitations. First, this study is qualitative with a small sample size, and the research results cannot be generalised to other groups or a wider area. Second, the research sample is concentrated on parents living in cities, and it is unclear whether there are differences in the caregiving experience of parents in rural areas, which may be affected by regional and cultural differences. In addition, this study focuses on the challenges faced by parents of children with ID in the process of care and their coping strategies regardless of their gender and roles. The inclusion of gender and roles could lead to different insights of the findings. It is recommended that future study should consider different methods, inclusion of gender and roles and wider geographical samples.

## Conclusion

This study concluded that parents of children with ID encounter four challenges, including emotional and psychological, economic, social, and core disorders in children. However, parents reported employing various coping strategies, such as self-regulation, continuous self-learning and personal growth, engagement in outdoor activity therapy, and actively seeking external support.

In-depth analysis of these challenges and coping strategies provides valuable insights for policymakers, social workers, and related professionals in designing targeted support services. Furthermore, understanding the role of these coping strategies can inform the development of more appropriate interventions to alleviate parental stress. This study also contributes to a deeper understanding of the experiences of parents of children with ID in China, offering important implications for practice and policy.

## Data Availability

The data used in this study are restricted due to confidentiality reasons. For further inquiries, please email to corresponding author.
